# Cognitive Behavioral Therapy versus Eye Movement Desensitization and Reprocessing in Patients with Post-traumatic Stress Disorder: Systematic Review and Meta-analysis of Randomized Clinical Trials

**DOI:** 10.7759/cureus.3250

**Published:** 2018-09-04

**Authors:** Ali M Khan, Sabrina Dar, Rizwan Ahmed, Ramya Bachu, Mahwish Adnan, Vijaya Padma Kotapati

**Affiliations:** 1 Psychiatry Resident, University of Texas Rio Grande Valley, Harlingen, USA; 2 Psychiatry, Saint Elizabeth's Medical Center, Boston, USA; 3 Psychiatry, Liaquat College, Karachi, PAK; 4 Psychiatry, Northwell Zucker Hillside Hospital, New York, USA; 5 Center for Addiction and Mental Health, University of Toronto, Toronto, CAN; 6 Psychiatry, Manhattan Psychiatric Center, New York, USA

**Keywords:** eye movement desensitization and reprocessing (emdr), post-traumatic stress disorder (ptsd), cognitive behavioral therapy (cbt)

## Abstract

Background

Post-traumatic stress disorder (PTSD) is prevalent in children, adolescents and adults. It can occur alone or in comorbidity with other disorders. A broad range of psychotherapies such as cognitive behavioral therapy (CBT) and eye movement desensitization and reprocessing (EMDR) have been developed for the treatment of PTSD.

Aim

Through quantitative meta-analysis, we aimed to compare the efficacy of CBT and EMDR: (i) relieving the post-traumatic symptoms, and (ii) alleviating anxiety and depression, in patients with PTSD.

Methods

We systematically searched EMBASE, Medline and Cochrane central register of controlled trials (CENTRAL) for articles published between 1999 and December 2017. Randomized clinical trials (RCTs) that compare CBT and EMDR in PTSD patients were included for quantitative meta-analysis using RevMan Version 5.

Results

Fourteen studies out of 714 were finally eligible. Meta-analysis of 11 studies (n = 547) showed that EMDR is better than CBT in reducing post-traumatic symptoms [SDM (95% CI) = -0.43 (-0.73 – -0.12), p = 0.006]. However, meta-analysis of four studies (n = 186) at three-month follow-up revealed no statistically significant difference [SDM (95% CI) = -0.21 (-0.50 – 0.08), p = 0.15]. The EMDR was also better than CBT in reducing anxiety [SDM (95% CI) = -0.71 (-1.21 – -0.21), p = 0.005]. Unfortunately, there was no difference between CBT and EMDR in reducing depression [SDM (95% CI) = -0.21 (-0.44 – 0.02), p = 0.08].

Conclusion

The results of this meta-analysis suggested that EMDR is better than CBT in reducing post-traumatic symptoms and anxiety. However, there was no difference reported in reducing depression. Large population randomized trials with longer follow-up are recommended to build conclusive evidence.

## Introduction

The definition of post-traumatic stress disorder (PTSD) has evolved over time and available criteria differ in many ways. According to the diagnostic and statistical manual of mental disorders (DSM-V), a traumatic event is defined as the exposure to actual or threatened death, serious injury or sexual violation and leading to re-experiencing, avoidance, negative cognitions and mood, and arousal [1]. But the ICD-10 Diagnostic Criteria for Research (ICD-10-DCR) only nominates events that lead to the distress of individuals [2]. The main variance between those two criteria is, the number of symptoms and duration of persistence. In addition, the DSM-V criteria require symptom persistence for more than a month whereas ICD-10-DCR necessitates symptom persistence for more than six months. The PTSD can occur due to natural or human events; natural incidents include natural calamity, accidents, assault, terrorist attacks, and war, while the human events include sexual assault, sudden death, kidnapping, serious illness and disaster [3].

Few studies have reported a comparison of occurrence of traumatic events among different countries. A comparison of 16 countries found Netherlands, Colombia and the USA had the highest rates of trauma exposure while the lowest rates of exposure were found in Italy, Romania and Spain [4]. However, not all people who exposed to trauma develop PTSD. Moreover, the risk of developing PTSD varies among different sections of the population. Population sections that are at a higher risk of developing PTSD are females, those lacking social support, economically marginalized, and the younger at time of exposure [5]. A survey of participants aged between 15 and 54 years in the United States found more than 60% of males and more than half of the females had experienced a traumatic event [6].

In the United States, the prevalence of PTSD has been estimated to be 8.3% [7]. Differences in prevalence of PTSD have documented in intentional and non-intentional trauma. Exposure to an intentional traumatic event increased the prevalence of PTSD over time while exposure to non-intentional events led to a decline in PTSD prevalence over time. In about 60% of individuals, traumatic symptoms resolve over time without any intervention. In case those patients developed PTSD, therapeutic options include psychotherapies and pharmacotherapies. Available psychotherapies are prolonged exposure, cognitive behaviour therapy (CBT) and eye movement desensitization and reprocessing (EMDR), while available pharmacotherapies are selective serotonin reuptake inhibitors (SSRI) [8].

The effectiveness of CBT in treating PTSD has been documented in many studies [9, 10]. In addition, the CBT has shown to be effective at reducing PTSD scores in PTSD associated with other conditions such as depression and anxiety. Similarly, the EMDR is one of the best remedies of PTSD as per World Health Organization (WHO) and there is a mounting number of studies reporting the effectiveness of EMDR in treating PTSD [11, 12]. EMDR has proved to be an effective treatment for reducing negative emotions and arousal. Additionally, the recent trials have established that EMDR has an effective role in reducing anxiety and depression in PTSD patients [3, 13].

Several recent meta-analyses have compared the efficacy of CBT and EMDR among children, adolescents and adults. A meta-analysis of 11 trials found that EMDR was slightly better than CBT in decreasing post-traumatic symptoms [14]. While another meta-analysis reported that EMDR and CBT were efficacious in reducing post-traumatic symptoms and depression but the reduction in depression was not statistically significant [15]. In contrast, the study by Seidler and Wagner did not find either EMDR or CBT to be superior at reducing post-traumatic symptoms [11]. Due to the contradictory results, we aimed to conduct an updated systematic review and meta-analysis to compare the efficacy of EMDR versus PTSD in reducing post-traumatic symptoms, anxiety, and depression.

## Materials and methods

Methods

This systematic review and meta-analysis is carried out in accordance with guidelines issued by Preferred Reporting for Systematic Reviews and Meta-analyses (PRISMA).

Literature search

Our systematic searching was limited to articles published between 1999 and December 2017. The process of identifying relevant articles began by searching for EMBASE, Medline and Cochrane Central Register of Controlled Trials. A manual search of the references list of included articles was conducted to identify any articles not retrieved by the database searches.

Inclusion and exclusion criteria

Only studies that met the following criteria were included in this meta-analysis: (1) study participants were children, adolescents or adults; (2) studies published in the English language; (3) studies that reported comparative results from randomized controlled trials of CBT and EMDR groups; (4) studies that reported a diagnosis of PTSD in accordance to DSM-V or DSM-IV or DSM-III; (4) studies that reported adequate data for calculation of design effect. The exclusion criteria were: (1) studies reporting treatment methodologies focused on conditions other than PTSD, such as depression, bipolar disorder, behavioral problems, substance abuse, psychosis, suicidal ideation, and substance dependence; (2) studies reporting other types of psychotherapies such as psychodynamic therapy, hypnotherapy, self-help, and biofeedback.

Search

The key terms used in the searching process met the guidelines of Medical Subject Heading Terms. The key words were: post-traumatic stress disorder, “PTSD”, eye movement desensitization and reprocessing, “EMDR”, Cognitive Behavioral Therapy, “CBT”. The Boolean connector AND was used to form different combinations of the keyword, see Table 1.

**Table 1:** Number of articles retrieved. EMDR: Eye movement desensitization and reprocessing; CBT: Cognitive behavioral therapy; PTSD: Post-traumatic stress disorder.

Key term	Number of articles in MEDLINE	Number of articles in EMBASE	Number of articles in Cochrane CENTRAL
Post-traumatic stress disorder AND EMDR and reprocessing AND CBT	35	29	2
PTSD AND EMDR AND CBT	36	46	8
PTSD AND eye movement desensitisation and reprocessing AND CBT	35	20	3
PTSD AND eye movement desensitisation and reprocessing AND Cognitive Behavioral Therapy	110	7	4
Post-traumatic stress disorder AND eye movement desensitisation and reprocessing AND Cognitive Behavioral Therapy	106	6	3
Post-traumatic stress disorder AND eye movement desensitisation and reprocessing AND CBT	34	20	2

Study selection

All articles identified from all the databases were imported into one Endnote library where all duplicates were removed. The unique study titles and abstracts were screened through two independent reviewers, to check if they met inclusion criteria. Studies that met the inclusion criteria were fully reviewed by a third contributor “Ramya Bachu”, to check if they reported the required data. Studies that did not report the required data were excluded.

Data extraction

Data extraction was carried out by two reviewers “Ali Khan” and “Padma Kotapati”. A third author “Ramya Bachu” compared the data to identify any inconsistencies. Any discrepancies were resolved through consultation among all authors, which ensured accurate data extraction process. For each included article, the means, standard deviations and p values of pre-treatment, post-treatment and follow-up were extracted. Post-traumatic symptoms were measured using the PTSD reaction index (PTSD-RI), children’s response to trauma index (CRTI), child report of post-traumatic symptoms (CROPS), clinician-administered PTSD scale child/adolescent version (CAPS-CA), clinician-administered PTSD scale (CAPS), and structured interview for PTSD (SI-PTSD). Anxiety was measured using hospital anxiety and depression scale (HADS), multidimensional anxiety scale for children (MASC), state-trait anxiety inventory (STAI), and revised children’s anxiety and depression scale (RCADS-C). Depression was measured using the Beck depression index (BDI). Other extracted variables were: age, gender, CBT variants, treatment fidelity, comorbidity, co-interventions, study duration, year of publication, and methodological characteristics useful in assessing study bias.

## Results

Literature search

**Figure 1:**
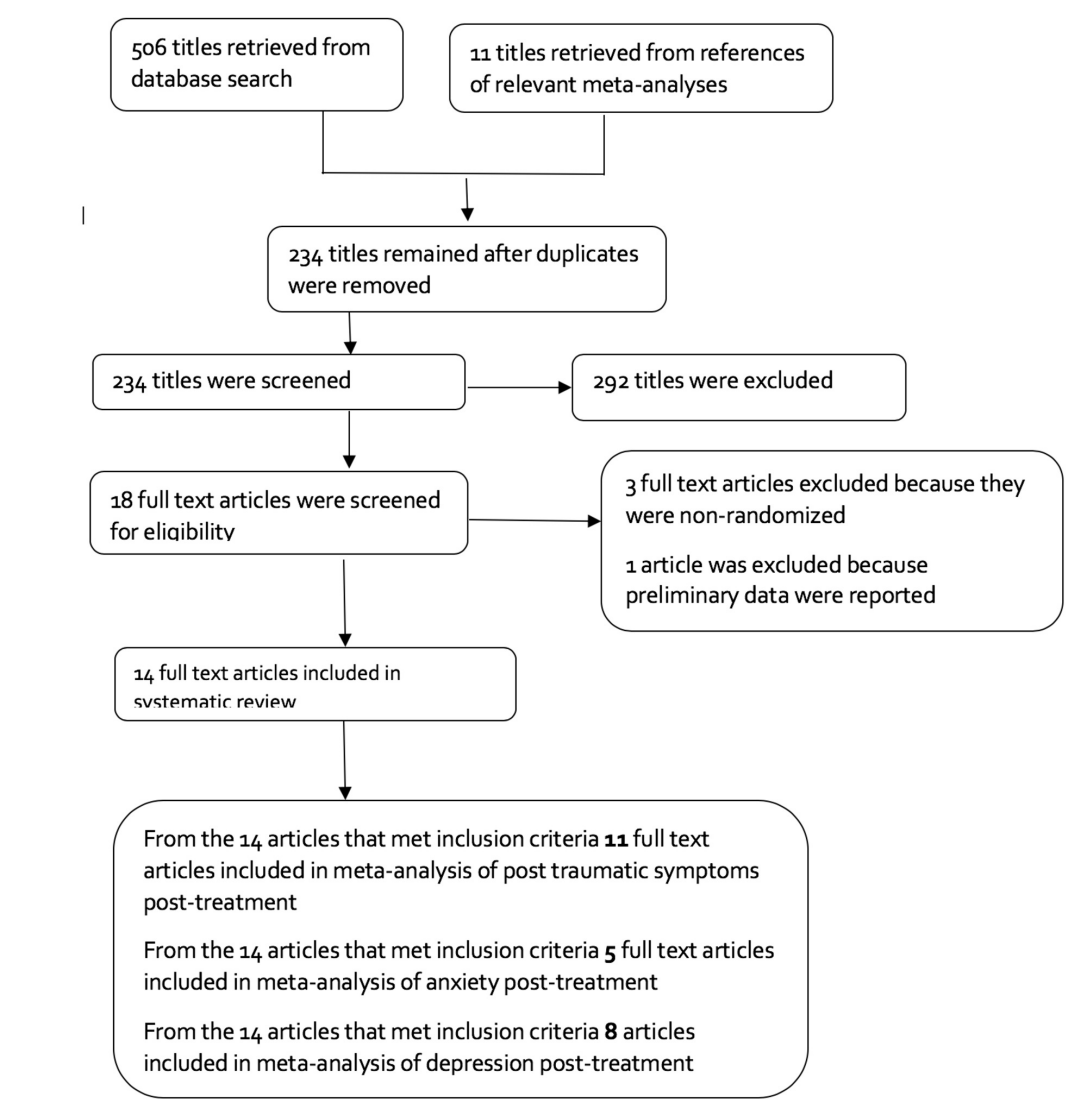
Selection of studies.

The number of articles found in each database through using of various key terms is shown in Table 1. Of all screened papers, 14 articles were finally eligible for meta-analysis. The flow diagram of studies’ selection is shown in Figure 1.

Baseline characters

The characteristics of included studies are shown in Table 2. The variants of CBT used were Imaginal Exposure (IE), Trauma Treatment Protocol (TTP), Prolonged exposure (PE), Trauma-Focused Cognitive Behavioral Therapy (TF-CBT), Stress Inoculation Training with Prolonged Exposure (SITPE), Exposure plus cognitive restructuring (E + CR), and brief eclectic psychotherapy. Some patients had comorbidities in two studies by van den Berg (2015) and de Roos (2017). Nine studies used “intent to treat” to minimize the impact of participants who dropped out. Eight studies used treatment fidelity to ensure adherence to the treatment protocol.

**Table 2:** Characteristics of included studies. N/A: Not applicable; PTSD: Post-traumatic stress disorder; CAPS: Clinician-administered PTSD Scale; PTSD-RI: PTSD-reaction index; CRTI: Children’s response to trauma index; CROPS: Child report of post-traumatic symptoms; PROPS: Parent report of post-traumatic stress symptoms; CAPS-CA: Clinician-administered PTSD scale child/adolescent version; SI-PTSD: Structured interview for PTSD; HADS: Hospital anxiety and depression scale; MASC: Multidimensional anxiety scale for children; STAI: State-trait anxiety inventory; RCADS-C: Revised children’s anxiety and depression scale; BDI: Becks depression inventory; IES-R: Impact of event scale-revised; BDS: Backward digit span; CBCL: Child behavior checklist; STAI-Y2: State-Trait Anxiety Inventory-Y2 Trait Form; SUD: Substance use disorder; CMS: The Mississippi Scale for Civilian PTSD; PSS-SR: PTSD symptom scale self-report version; CEQ: Combat experiences questionnaire; DEVS-T: Distress evaluation scale for treatment; DES: Dissociative experiences scale; CRIES 13: Children’s revised impact of event scale; RCADS: Revised child anxiety and depression scale; IOE: Impact of events scale; HADS: Hospital anxiety and depression score; CRTI: Children's responses to trauma inventory; C-PTCI: Children's post-traumatic cognitions inventory; RCADS- C/P: Revised children’s anxiety and depression scale- children/parent version; SDQ-A/P: Strengths and difficulties questionnaire; CSI-C/P: Coping strategies inventory- Child/Parent; DES II: Dissociative experiences scale-II; QPF-R: The psychophysiological questionnaire-brief version; SCL-90 = R: Symptom checklist-90-Revised; SLESQ: Stressful life events screening questionnaire; SCID: Structured clinical interview for DSM-5; ADIS-C/P: Anxiety disorder interview schedule for DSM-IV: Child and parent interview schedule; MADRS: Montgomery Åsberg depression rating scale; MMPI-K: Minnesota multiphasic personality inventory-K scale; TF-CBT: Trauma-focused cognitive behavioral therapy; HAM-A: Hamilton anxiety rating scale; CAPS-CA: Clinician-administered PTSD scale based upon DSM-5;* *PPD: Postpartum depression.

Study	Variants of CBT	Trauma type	Clinician-rated measures	Self-rated measures	Mean age (years)	Males/Females	Months post	Intention-to-treat (ITT) analysis	Treatment fidelity check	Comorbidity
Arabia et al., 2011 [13]	Imaginal Exposure	N/A		IES-R, STAI, BDI	63.48	28/14	6	Yes	Yes	Not reported
De Roos et al., 2011 [16]				PTSD-RI, CROPS, PROPS, BDS, MASC, CBCL	10.1	29/23	3	Yes	No	Not reported
Devilly et al., 1999 [17]	Trauma Treatment Protocol	Mixed		STAI-Y2, BDI, SCL-90=R, SUD, PPD, CMS, IES, PSS-SR, CEQ, DEVS-T	37.96	8/15	3	No	Yes	Not reported
Ironson et al., 2002 [18]	Prolonged Exposure	Mixed		PSS-SR, BDI, DES, SUD		5/17	3	No	Yes	Not reported
Jaberghaderi et al., 2004 [19]	Trauma Focused-CBT	Sexual assault	Rutter teacher scale	CROPS, PROPS	12.5	0/14		No	No	Not reported
Diehle et al., 2015 [3]		Mixed	CAPS-CA	CRIES 13, RCADS,	12.9	18/30		Yes	Yes	Not reported
Lee et al., 2002 [20]	Stress Inoculation Training and Prolonged Exposure	Mixed	SI-PTSD, MMPI-K,	IES, BDI	34.0	13/11	3	No	Yes	Not reported
Power et al., 2002 [21]	Exposure + Cognitive Restructuring	Mixed	CAPS, MADRS, HAM-A	IOE, SI-PTSD, HADS, Sheehan disability index	40.9	42/30	15	No	No	Not reported
De Roos et al., 2017 [22]			ADIS-C/P	CRTI, C-PTCI, RCADS-C/P, SDQ-A/P, CSI-C/P	13.06	44/59	12	Yes	Yes	Anxiety disorders
Rothbaum et al., 2005 [12]	Prolonged Exposure	Sexual assault	CAPS, SLESQ, SCID non-patient version	PSS-SR, IES-R, BDI, DES-II, STAI	33.8	0/60	6	Yes	No	Not reported
Taylor et al., 2003 [23]	Exposure	Mixed	CAPS	BDI	37	15/45	3	Yes	No	Not reported
Nijdam et al., 2012 [24]	Brief eclectic psychotherapy	N/A		IES-R, SI-PTSD, SCID-I, HADS				Yes	Yes	Not reported
Capezzani et al., 2013 [25]			CAPS	BDI, STAI-Y, QPF-R, IES-R				No	No	Not reported
Van den Berg et al., 2015 [26]	Prolonged Exposure	Mixed	CAPS	PSS-SR, PTCI	41.2	84/71	6	Yes	Yes	Psychotic disorder

Quality assessment

All studies were assessed for risk of bias through the Cochrane tool. The risk of bias was defined as low, medium, high, and unclear. All studies reported random sequence allocation. There was a high risk of bias in four studies regarding blinding of the outcome. Only four studies reported allocation concealment. Detailed risk of bias assessment is shown in Table 3.

**Table 3:** Detailed risk of bias assessment. EMDR: Eye movement desensitization and reprocessing; IE: Imaginal exposure; ITT: Intention to treat; CBT: Cognitive behavioral therapy; TTP: Trauma treatment protocol; PE: Prolonged exposure; SITPE: Stress inoculation training with prolonged exposure; E+ CR: Exposure plus cognitive restructuring; WL: Waiting list; WAIT: No treatment wait list control.

Domain	Allocation concealment		Blinding of outcome assessment		Incomplete outcome data		Selective reporting	
Study	Judgment	Support for judgment	Judgment	Support for judgment	Judgment	Support for judgment	Judgment	Support for judgment
Arabia et al., 2011 [13]	Unclear	Not reported	Unclear	Not reported	Low risk	Five participants in EMDR and three participants in IE lost to follow up at month 6. ITT was used.	Low risk	All measures were reported
De Roos et al., 2011 [16]	Unclear	Not reported	Low risk	Assessor was blinded to treatment conditions	Low risk	Eight participants in EMDR group dropped out and six participants in CBT group dropped out. ITT and imputation used to account for missing observations	Low risk	All measures were reported
Devilly and Spence, 1999 [17]	Unclear	Not reported	Unclear	Not reported	High risk	Three participants in TTP group dropped out and six participants in EMDR group dropped out. Impact of drop out was not discussed	Low risk	All measures were reported
Ironson et al., 2002 [18]	Unclear	Not reported	High risk	Assessors were not blinded to treatment conditions		Three participants in PE dropped out and there was no drop out in EMDR	Low risk	All measures were reported
Jaberghaderi et al., 2004 [19]	Unclear	Not reported	Low risk	Screening was done by psychologists blinded to treatment	Low risk	One participant in EMDR and one participant in CBT dropped out. This was a low drop out	Low risk	All measures were reported
Diehle et al., 2015 [3]	Low risk	A researcher not involved in study managed randomization list and communicated to therapist	Low risk	Assessors were blinded to treatment	Low risk	12 participants lost to follow up. ITT and imputation were used to handle missing observations	Low risk	All measures were reported
Lee et al., 2002 [20]	Unclear	Not reported	High risk	Assessor was blinded at pre-treatment but not at post-treatment and follow-up	Low risk	One participant from SITPE and one participant from EMDR dropped out. This was a minimal drop out	Low risk	All measures were reported
Power et al., 2002 [21]	Low risk	Sealed envelope was used to conceal randomization group	Medium risk	Pre- and post-treatment assessors were blinded but there was no blind mid-point and at follow-up	Medium risk	12 participants in EMDR dropped out, 16 in E+CR and five in WL. Impact of dropped participants not clear	Low risk	All measures were reported
De Roos et al., 2017 [22]	Low risk	Opaque sealed envelopes containing cards with trial arms were used to conceal allocation	Low risk	Independent assessors were blinded to treatment	Unclear	Not reported	Low risk	All measures were reported
Rothbaum et al., 2005 [12]	Unclear	Not reported	Low risk	Assessors were blinded to treatment	Low risk	12 participants dropped out of the study. PE = 3, EMDR = 5, WAIT = 4. ITT did not provide different results.	Low risk	All measures were reported
Taylor et al., 2003 [23]	Unclear	Not reported	Low risk	Interviewers were blinded to treatment		Five patients in EMDR and seven patients in PE dropped out.	Unclear	Not reported
Nijdam et al., 2012 [24]	Low risk	A psychologist not involved in study had randomization file	Low risk	Assessors were masked to treatment group	High risk	20 participants in EMDR and 25 participants in brief eclectic therapy dropped out. Although ITT was used this was a significant drop out		
Capezzani et al., 2013 [25]	Unclear	Not reported	Low risk	CAPS was administered by blind assessor	Low risk	No patient dropouts	Low risk	All measures were reported
Van den Berg et al., 2015 [26]	Unclear	Not reported	Low risk	There was blinding but 27 incidences of un-blinding occurred and these assessments were repeated	Low risk	13 participants in PE and 11 participants in EMDR dropped out. Completer analyses and ITT did not yield different results	Unclear	BDI scores not reported post-treatment and at six month follow-up

Meta-analysis of post-traumatic symptoms post-treatment

**Figure 2:**
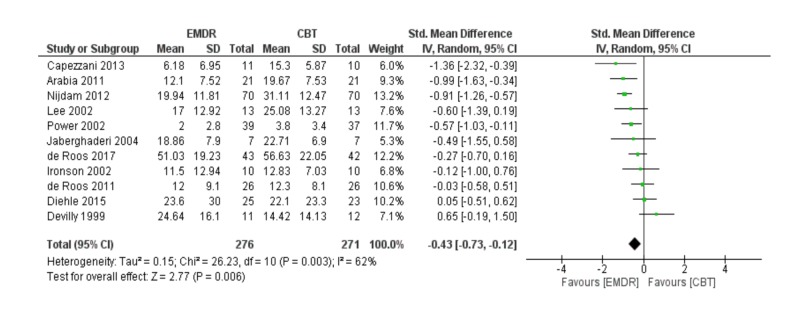
Meta-analysis of post-traumatic symptoms post-treatment. EMDR: Eye movement desensitization and reprocessing; CBT: Cognitive behavioral therapy.

**Figure 3:**
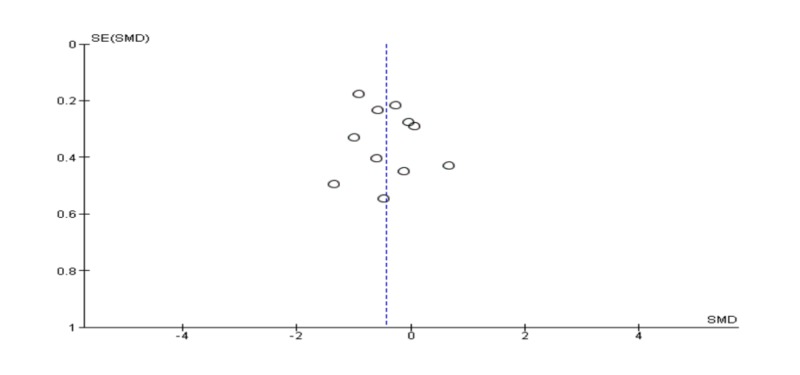
Funnel plot for meta-analysis of post-traumatic symptoms post-treatment.

Pooling 11 studies in a meta-analysis, the EMDR was better than CBT in reducing post-traumatic symptoms [SDM (95% CI) = -0.43 (-0.73 – -0.12)]. The results were statistically significant (p = 0.006); however, the studies included in this quantitative meta-analysis had a high level of heterogeneity (I2 = 62%) mentioned in Figure 2. Also, a funnel plot of publication bias did not show any asymmetry and no bias among included studies mentioned in Figure 3.

Meta-analysis of PTSD symptoms at three months follow-up

**Figure 4:**
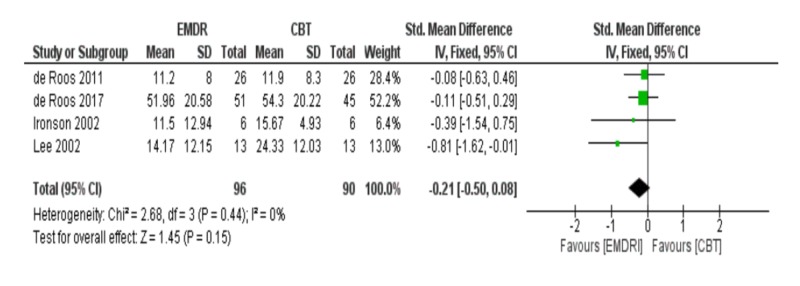
Meta-analysis of PTSD symptoms at three months follow-up. EMDR: Eye movement desensitization and reprocessing; CBT: Cognitive behavioral therapy; PTSD: Post-traumatic stress disorder.

**Figure 5:**
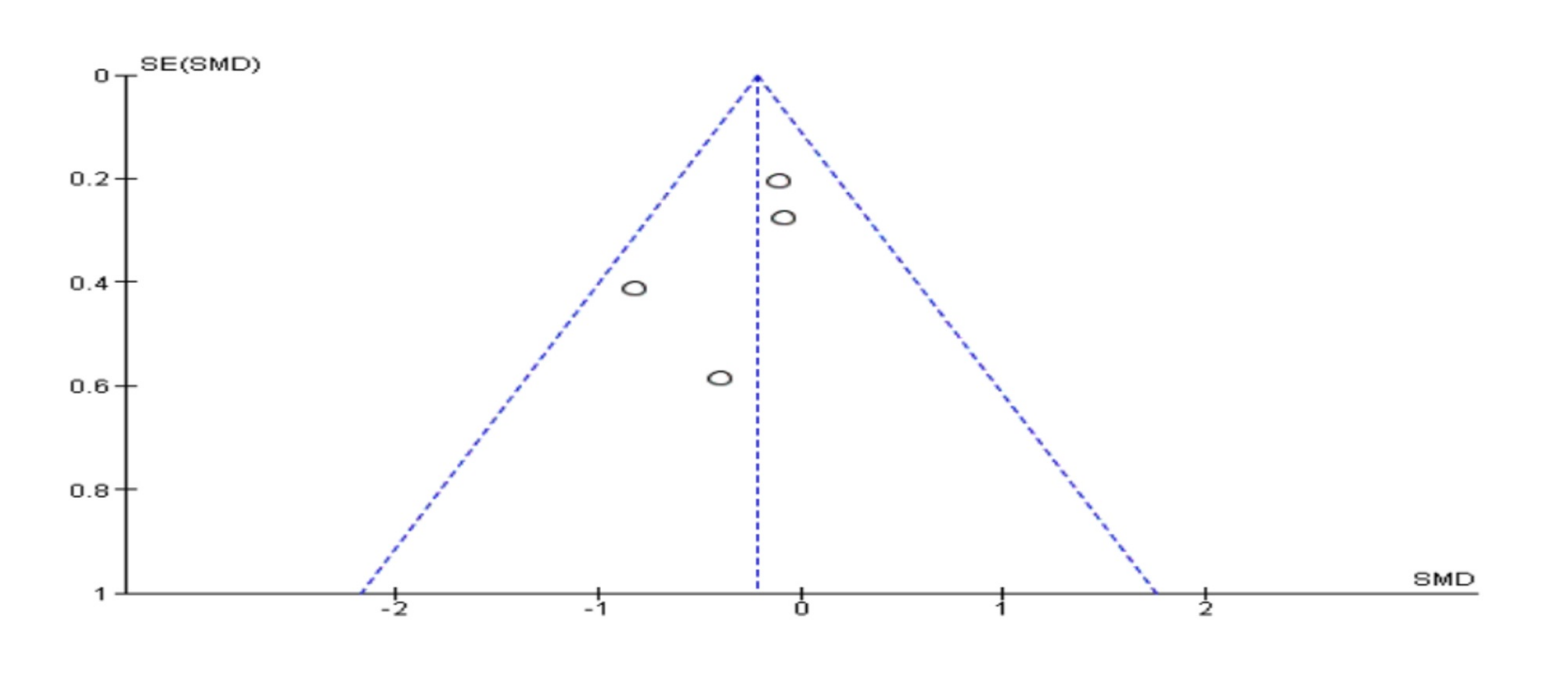
Funnel plot of meta-analysis of PTSD symptoms at three months follow-up. PTSD: Post-traumatic stress disorder.

At three months follow-up, the meta-analysis of four studies showed that EMDR was not better than CBT in reducing post-traumatic symptoms [SDM (95% CI) = -0.21 (-0.50 – 0.08), p = 0.15] mentioned in Figure 4. Also, a funnel plot of publication bias of this meta-analysis did not show any asymmetry and no bias among included studies in Figure 5.

Meta-analysis of anxiety post-treatment

**Figure 6:**
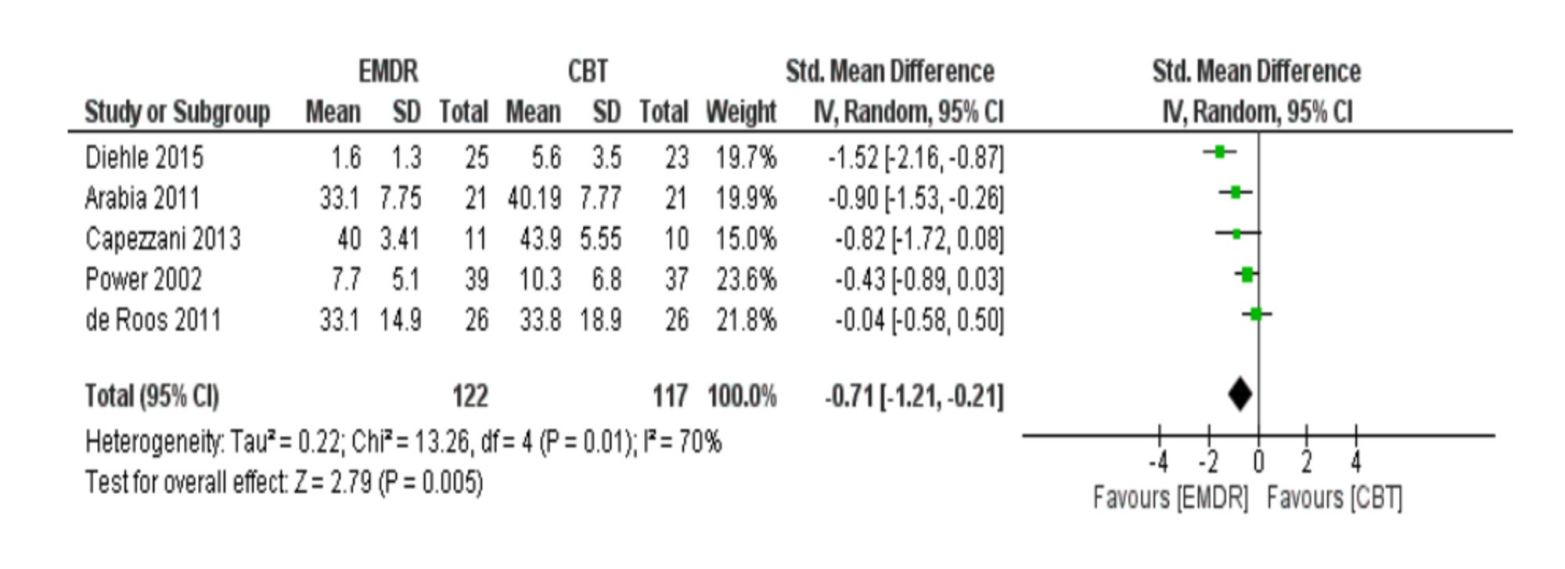
Meta-analysis of anxiety post-treatment. EMDR: Eye movement desensitization and reprocessing; CBT: Cognitive behavioral therapy.

**Figure 7:**
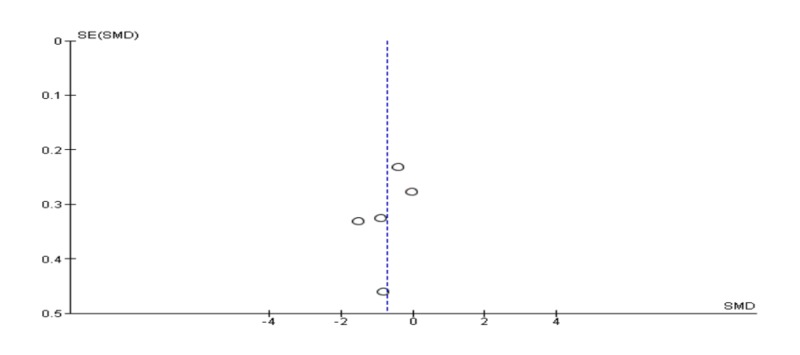
Funnel plot of meta-analysis of anxiety post-treatment.

The meta-analysis of five studies including 239 patients revealed that EMDR is better than CBT in reducing anxiety symptoms [SDM (95% CI) = -0.71 (-1.21 – -0.21)]. The result was statistically significant (p = 0.005) but there was marked heterogeneity between included studies (I2 = 70%) in Figure 6. Of note, there was no evidence of publication bias in the included studies in Figure 7.

Meta-analysis of depression post-treatment

**Figure 8:**
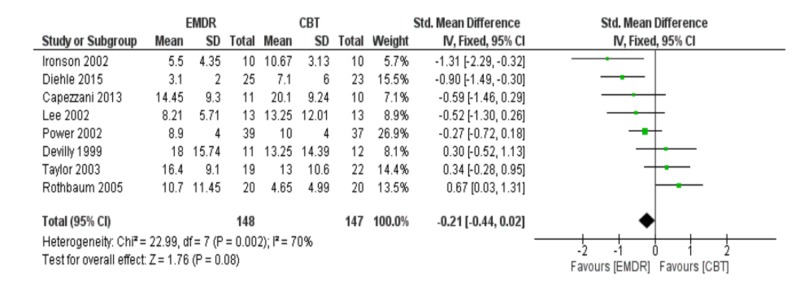
Meta-analysis of depression post-treatment. EMDR: Eye movement desensitization and reprocessing; CBT: Cognitive behavioral therapy.

**Figure 9:**
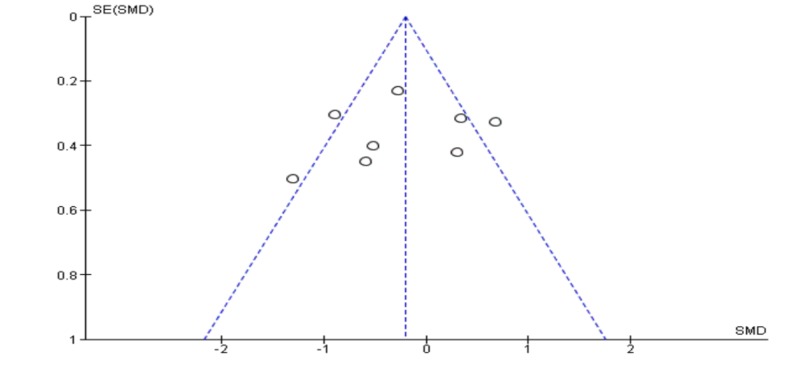
Funnel plot of meta-analysis of depression post-treatment.

The meta-analysis of depression symptoms was based on eight studies. The meta-analysis showed that EMDR was not better than CBT in reducing depression [SDM (95% CI) = -0.21 (-0.44 – 0.02), p = 0.08]. Also, the included studies had a high incidence of heterogeneity (I2 = 70%). These results are shown in Figure 8. Of note, there was no evidence of publication bias in the included studies in Figure 9.

Meta-analysis of depression at three months follow-up

**Figure 10:**
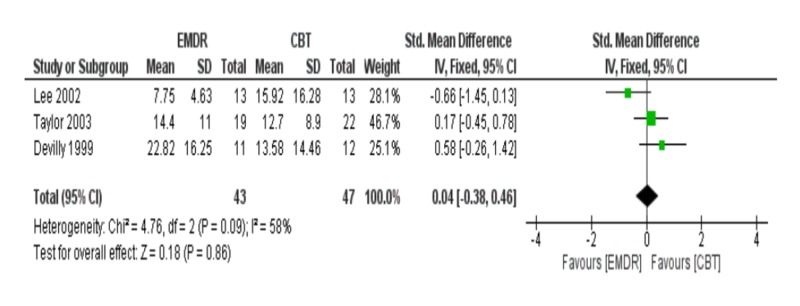
Meta-analysis of depression at three months follow-up. EMDR: Eye movement desensitization and reprocessing; CBT: Cognitive behavioral therapy.

**Figure 11:**
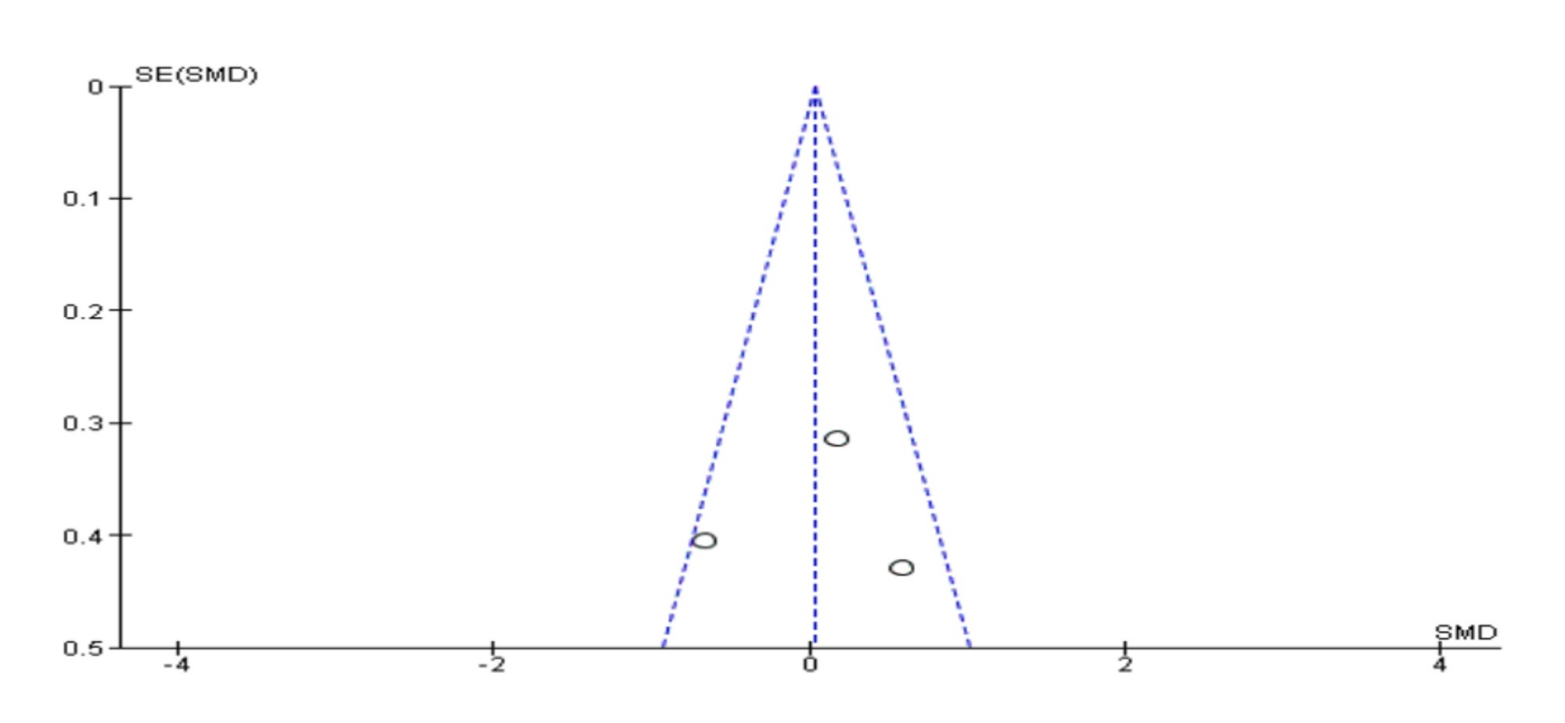
Funnel plot of meta-analysis of depression at three months follow-up.

Meta-analysis of three studies at three months follow-up showed that EMDR was not superior to CBT in reducing depression symptoms [SDM (95% CI) = 0.04 (-0.38 – 0.46), p = 0.86]. Also, the included studies had a high incidence of heterogeneity (I2 = 58%) in Figure 10. No evidence of publication as shown in the funnel plot in Figure 11.

## Discussion

The objectives of this meta-analysis were to compare the efficacy of EMDR and CBT in alleviating post-traumatic symptoms, anxiety and depression. The key result of this meta-analysis is, participants treated with EMDR had better alleviation of post-traumatic symptoms as compared to participants treated with CBT (p = 0.006). However, the superiority of EMDR at three months follow-up was not evident. The EMDR also had a statistically significant superiority over CBT (p = 0.005) in alleviating anxiety. Although EMDR was observed to be better than CBT in reducing depression, this difference was not statistically significant.

These findings have similarities and differences with the findings of other meta-analyses comparing EMDR and CBT. A meta-analysis by Moreno-Alcazar et al. found that EMDR was better than CBT in reducing post-traumatic symptoms among children and adolescents (p = 0.013) [27]. However, the difference detected by Moreno-Alcazar et al., d = -0.49, was higher than the difference between EMDR and CBT regarding reducing post-traumatic symptoms detected in the current meta-analysis. Similarly, a study by Chen et al. showed that EMDR was better than CBT in alleviating post-traumatic symptoms (p = 0.05) [28]. The difference found by Chen et al. was exactly the same as the difference determined in the current analysis, but the current analysis had stronger evidence of statistical significance. Also, the current analysis had lower heterogeneity (I2 = 62%) as compared to a meta-analysis by Chen et al. (I2 = 70%). In the current analysis, EMDR was found to be better than CBT post-treatment; however, the study by Seidler and Wagner did not detect any superiority [11]. This difference could probably be attributed to the inclusion of more randomized trials in our recent meta-analysis compared to their study conducted in 2006. Nevertheless, at three months follow-up, the findings of the current meta-analysis were consistent with the findings of Seidler and Wagner.

The results of the meta-analysis by Lee and Ho (2012) and Davidson and Parker (2001) found inconsistent results with the current meta-analysis on the efficacy of EMDR and CBT [29, 30]. Their meta-analysis did not find a significant difference between EMDR and CBT (p = 0.085) in alleviating post-traumatic symptoms while the current meta-analysis found a statistically significant difference. It is noteworthy mentioning that the Davidson and Parker (2001), Seidler and Wagner (2006), and Lee and Ho (2012) only included studies that have been published up to 2006. While our study aimed to investigate studies published before and after 2006, which will remain an important advantage of our meta-analysis compared to previous studies [30,11,29].

Our meta-analysis declared no difference between CBT and EMDR post-treatment in alleviating depression, which is incompatible with the findings of Seidler and Wagner (2001) who found that EMDR was better than CBT in reducing depression symptoms. Of note, the meta-analysis by Seidler and Wagner included seven studies while the current analysis meta-analyzed eight studies. Additionally, in agreement with the findings of Seidler and Wagner (2001), the meta-analysis by Ho and Lee (2012) found that the EMDR was better than TF-CBT in alleviating depression. In contrast, the concept that no difference between EMDR and CBT in alleviating depression was consistent with the findings of Moreno-Alcazar et al. (2012) who did not find a statistically significant difference.

Regarding reducing anxiety, the current meta-analysis reported that EMDR was better than CBT in alleviating anxiety, which is consistent with the findings of Moreno-Alcazar et al. (2017) who also found a statistically significant difference.

When translating the findings of this meta-analysis into clinical practice, there are several limitations that need to be considered. For instance, there are few numbers of included participants in the analyzed randomized trials. We also searched only three major databases; however, we considered the manual searching to cover more articles. The subgroup meta-analysis of post-traumatic symptoms and depression at three months follow-up, comprised a few number of analyzed studies. Our inferences must be interpreted cautiously due to the presence of bias in more than one domain of included trials. We also did not conduct meta-regression of various variables such as age and sex due to a small number of included studies. Future large-scale randomized trials are warranted with longer follow-up periods and adherence to well-designed protocols.

## Conclusions

The results of this meta-analysis suggested that EMDR is better than CBT in reducing post-traumatic symptoms and anxiety. However, there was no difference reported in reducing depression. Large population randomized trials with longer follow-up are recommended to build conclusive evidence.
